# Design, Synthesis, and Investigation of Cytotoxic Effects of 5-Hydroxyindole-3-Carboxylic Acid and Ester Derivatives as Potential Anti-breast Cancer Agents

**DOI:** 10.5812/ijpr-133868

**Published:** 2023-04-17

**Authors:** Arezo Teymori, Shaya Mokhtari, Anna Sedaghat, Arash Mahboubi, Farzad Kobarfard

**Affiliations:** 1 Department of Medicinal Chemistry, School of Pharmacy, Shahid Beheshti University of Medical Sciences, Tehran, Iran; 2 Central Research Laboratories, Shahid Beheshti University of Medical Sciences, Tehran, Iran; 3 Phytochemistry Research Center, Shahid Beheshti University of Medical Sciences, Tehran, Iran; 4 Food Safety Research Center, Shahid Beheshti University of Medical Sciences, Tehran, Iran

**Keywords:** 5-Hydroxy Indole, Survivin, Human Breast Cancer Cell Line (MCF-7), MTT Assay

## Abstract

Breast cancer is a deadly disease with a high prevalence rate among females. Despite several treatments, scientists are still engaged in finding less invasive treatments for this disease. The cellular proliferation rate and cell viability survey are critical to assess the drug’s effect on both normal and malignant cell populations. Indole derivatives are promising candidates for their cytotoxic effect causing on breast cancer cells; however, they are less toxic on normal cells. This study synthesized 23 novel 5-hydroxyindole-3-carboxylic acids and related esters featuring various linear, cyclic, and primary aromatic amines. The MTT assay indicated the cytotoxicity of all acid and ester derivatives against the MCF-7 cells with no significant cytotoxicity on normal human dermal fibroblasts cells. Compound 5d, an ester derivative possessing a 4-methoxy group, was the most potent compound, with a half-maximal effective concentration of 4.7 µM. Compounds 5a, 5d, and 5l bearing ester group in their structure demonstrated cytotoxicity values < 10 µM against the MCF-7 cell line and were safe for advanced screening.

## 1. Background

**Figure 1.**
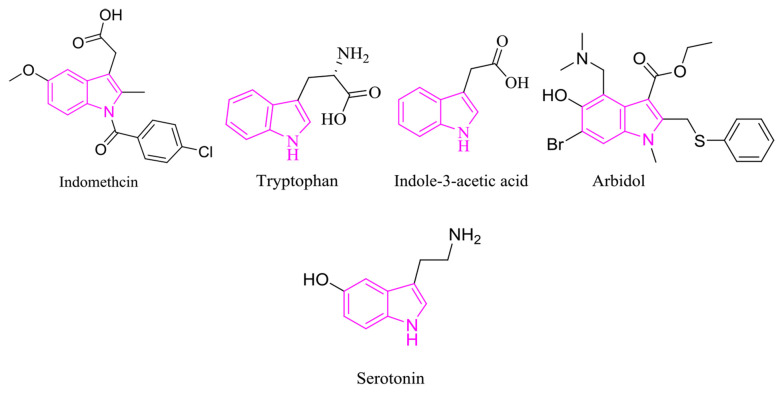
Natural and medicinal compounds bearing indole scaffold

Breast cancer is one of the most well-known cancers in females. Due to its effect on the population, this disease presents a critical health problem that requires further molecular investigation to specify its prognosis and treatment (1). Indole derivatives’ diverse abilities and unique physicochemical properties make them an ideal scaffold in drug design; accordingly, their footprint can be found in many natural and synthetic therapeutic agents. Tryptophan (i.e., an essential amino acid), serotonin (i.e., a polyfunctional signaling molecule) (2-5), indole-3-acetic acid (i.e., a plant hormone), indomethacin (i.e., a nonsteroidal anti-inflammatory drug), arbidol (i.e., an antiviral) are only a few famous examples. Indoles exhibit diverse pharmacological activities, primarily highlighting their anti-cancer effects, followed by antimicrobial, antimalarial, and antituberculosis effects (Figure 1) (6-10).

In the present study, novel indole derivatives were synthesized and screened for cytotoxicity against MCF-7 (Michigan Cancer Foundation-7) cells using the MTT assay and compared to cisplatin as a commonly used chemotherapeutic agent in treating many tumors (11). Recent investigations have proven that indolic compounds do not adversely affect the kidney and liver (12). Additionally, in one study, indomethacin, containing an indole scaffold, was observed as an effective agent, with an unknown mechanism, to inhibit breast cancer (13).

**Figure 2.**
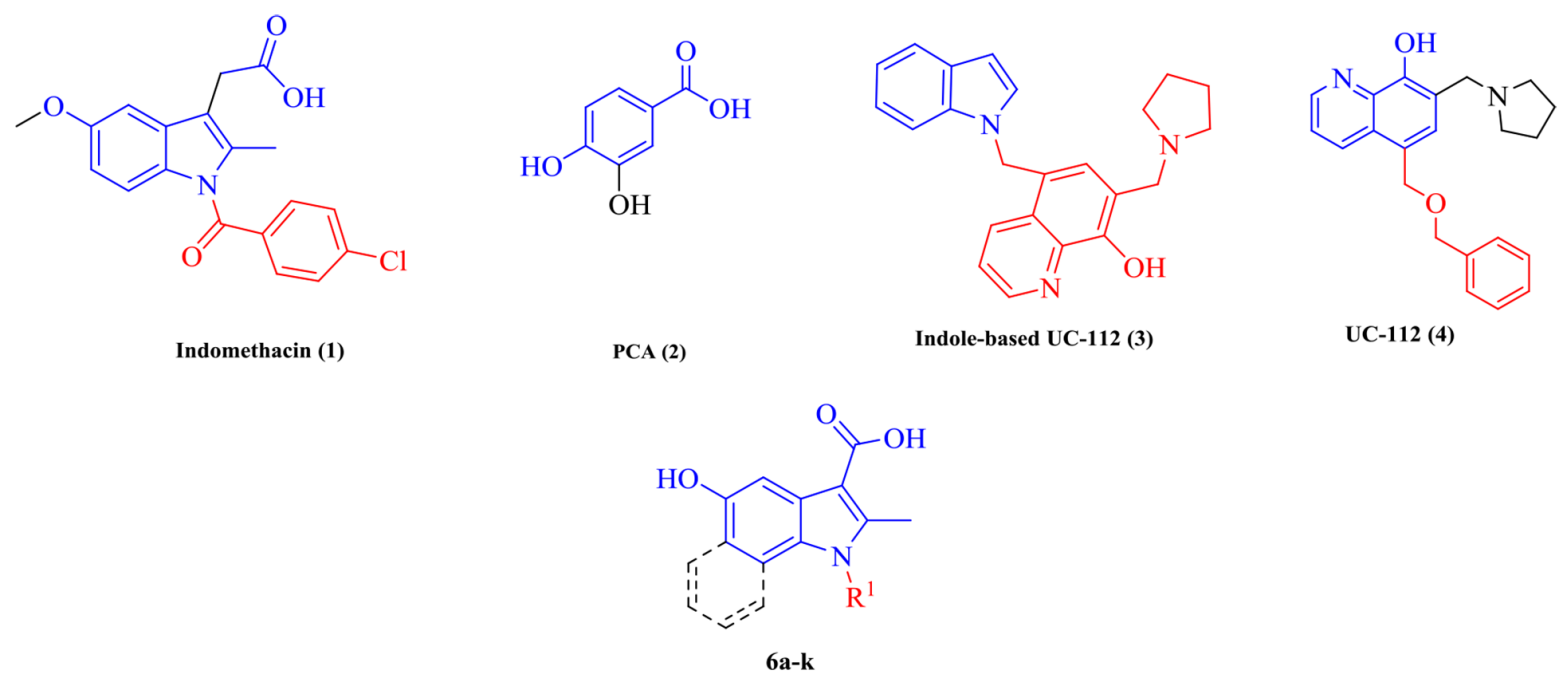
Structure of lead compounds (1-4) and the present study’s designed structures (6a-k)

Another study claimed that indomethacin is a survivin inhibitor (14). Survivin was detected as the smallest member of the inhibitors of apoptosis (IAPs) protein family. Survivin in aggressive tumors by binding to caspase-3 and 7 inhibits apoptosis (15, 16). UC-112 is a small molecule that strongly inhibits cancer cell proliferation and selectively degrades survivin among other IAPs. Indole analogs of UC-112 as selective and effective survivin inhibitors indicate that indole is an essential pharmacophore in survivin inhibitory agents (17, 18). On the other hand, protocatechuic acid (PCA), a plant phenolic acid, inhibits proliferation and induces apoptotic effects on breast cancer. Through the survivin inhibition mechanism, apoptosis removes malignant cells without causing damage to normal cells or tissues (19-21). The present study applied molecular hybridization techniques to design new compounds based on indomethacin, UC-112, and PCA structures. In the design of the target molecules (6a-6k), the -CH_2_-COOH group in indomethacin was replaced by -COOH or -COOEt group, and different *N*-substituted indole cores bearing a 5-OH group to resemble PCA were synthesized (Figure 2).

## 2. Methods

### 2.1. General

Reagents and chemicals used were purchased from Merck Corporation (USA). Melting points were measured by an Electrothermal 9100 device. Cary 630 FTIR spectrometer recorded infrared spectra in potassium bromide (KBr) in υ_max_ (cm^-1^). Bruker 400 Avance apparatus, in dimethyl sulfoxide (DMSO)-d_6_ at 400.1 and 100 MHz, measured ^1^H-NMR and ^13^C-NMR. HPLC 6410 Agilent device measured liquid chromatography (LC) mass spectra and the Costech (Italy) elemental analyzer measured elemental analysis. ChemDraw Professional software (version 16) was used to measure acids and esters’ log P. Discovery studio 4.5 visualizer, a feature-rich molecular modeling application, was used for the final docking image of a 5d small molecule with survivin protein.

### 2.2. General Experimental Approach

**Figure 3.**
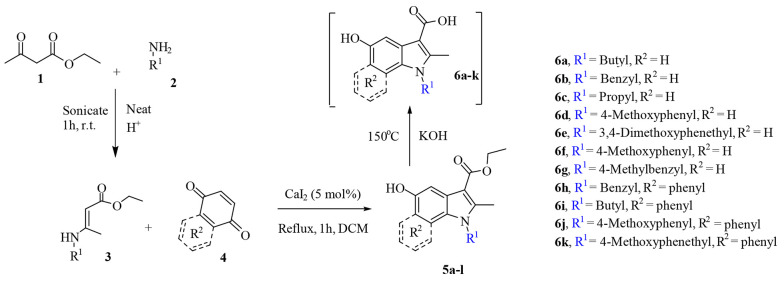
Synthesis of 5-hydroxyindole-carboxylic acid derivatives

The process consists of three steps, namely synthesis of enamine, 5-hydroxyindoles carboxylic ester synthesis, and basic ester cleavage of 5-hydroxyindoles carboxylic ester (Figure 3). The primary enamine was obtained under ultrasonic irradiation with the catalytic amounts of acetic acid in a round-bottom flask in which ethyl acetoacetate 1 and primary amine 2 were added. Then, enamine 3 was added dropwise to benzoquinone or naphthoquinone 4 in the presence of CaI_2_ as a catalyst and reacted for one hour at reflux temperature, and the product (i.e., 5-hydroxyindoles carboxylic ester 5) was obtained, which undergoes subsequent hydrolysis to form 5-hydroxyindoles carboxylic acid 6.

#### 1-Butyl-5-Hydroxy-2-Methyl-1H-Indole-3- Carboxylic Acid (6a)

Cream powder; yield: (85%); mp: 129 - 130°C. IR (ν_max_, cm^-1^): 3283, 2959, 1647, 1245, 868. ^1^H-NMR (DMSO-d6-400MHz) *δ*_H_ = 8.82 (s, 1H, OH), 7.24 (d, *J* = 8.4 Hz, 1H, Ar), 7.12 (d, *J* = 2 Hz, 1H, Ar), 6.64 (dd, *J* = 8.4, 2 Hz, 1H, Ar), 4.08 (t, *J* = 7.2 Hz, 2H, CH_2_N), 2.54 (s, 3H, Me), 1.63 (m, 2H, CH_2_), 1.37 - 1.24 (m, 2H, CH_2_), 0.89 (t, *J* = 7.6 Hz, 3H, Me). ^13^C-NMR (DMSO-d6-100MHz) *δ*_C_ = 166.83 (C=O), 152.10 (C), 139.57 (C=N), 130.27 (C=N), 126.76 (C), 111.30 (C), 110.50 (CH), 106.59 (CH), 104.75 (CH), 42.71 (CH_2_N), 32.15 (CH_2_), 20.02 (CH_2_), 14.18 (CH_3_), 11.88 (CH_3_) LC-MS (ESI) m/z 270.12 (M+Na^+^).

#### 1-Benzyl-5-Hydroxy-2-Methyl-1H-Indole-3-Carboxylic Acid (6b)

White powder; yield: (86%); mp: 143 - 145°C. IR (KBr) (ν_max_, cm^-1^): 3302, 3063, 1666, 1297, 838. ^1^H-NMR *δ*_H_ = 8.99 (s, 1H, OH), 7.43 (s, 1H, Ar), 7.28 (d, *J* = 11.6 Hz, 1H, Ar), 7.12 - 6.9 (m, 5H, Ar), 6.67 (d, *J* = 11.6 Hz, 1H, Ar), 5.38 (s, 2H, CH_2_N), 2.66 (s, 3H, Me). ^13^C-NMR *δ*_C_ = 165.60 (C=O), 153.25 (C), 138.10 (C), 137.60 (C=N), 132.93 (C=N), 131.70 (C=C), 130.81 (C), 129.71 (C), 127.69 (C=C), 126.62 (C=C), 125.60 (C=C), 112.05 (C), 111.26 (C=C), 106.02 (C=C), 103.10 (C=C), 59.23 (CH_2_N), 12.28 (CH_3_). LC-MS (ESI) m/z 282 (M+H^+^). Anal. Calc. for C_17_H_15_NO_3_: C, 72.58; H, 5.37; N, 4.98. Found: C, 72.54; H, 5.39; N, 5.00.

#### 5-Hydroxy-2-Methyl-1-Propyl-1H-Indole-3-Carboxylic Acid (6c)

Light blue powder; yield: (80%); mp: 120 - 123°C. IR (ν_max_, cm^-1^): 3302, 1602, 1233, 820. ^1^H-NMR: *δ*_H_ = 8.99 (s, 1H, OH), 7.25 (d, *J* = 8.4 Hz, 1H, Ar), 6.63 (dd, *J* = 8.4, 1.6 Hz, 1H, Ar), 4.28 (t, *J* = 7.2 Hz, 2H, CH_2_), 2.54 (s, 3H, Me), 1.67 (q, *J* = 7.2 Hz, 2H, CH_2_), 0.88 (t, *J* = 7.2 Hz, 3H, Me). ^13^C-NMR *δ*_C_ = 166.83 (C=O), 152.08 (C), 139.63 (C=N), 130.34 (C=N), 126.75 (C), 111.29 (C), 110.56 (CH), 106.58 (CH), 104.73 (CH), 44.38 (CH_2_N), 23.52 (CH_2_), 12.94 (CH_3_), 11.58 (CH_3_). LC-MS (ESI) m/z 235 (M+H^+^).

#### 5-Hydroxy-1-(4-Methoxyphenyl)-2-Methyl-1H-Indole-3-Carboxylic Acid (6d)

Cream powder; yield: (83%); mp: 189 - 191°C. IR (KBr) (ν_max_, cm^-1^): 3313, 2985, 1710, 1646, 1252, 864. ^1^H-NMR *δ*_H_ = 7.47 (d, *J* = 4 Hz, 1H, Ar), 7.36 - 7.34 (d, *J* = 8 Hz, 2H, Ar), 7.15 - 7.13 (d, *J* = 8 Hz, 2H, Ar), 6.74 (d, *J* = 8 Hz, 1H, Ar), 6.60 (dd, *J* = 8, 4Hz 1H, Ar), 3.85 (s, 3H, OMe), 2.45 (s, 3H, Me). ^13^C-NMR *δ*_C_ = 167.30 (C=O), 159.58 (C), 153.28 (C), 145.26 (C), 132.20 (C=N), 129.65 (2C), 129.17 (C=N), 127.94 (C), 115.38 (2CH), 112.13 (C), 111 (CH), 105.97 (CH), 104.52 (CH), 55.93 (CH_3_O), 13.24 (CH_3_). MS: m/z (%) = 298 (M+H^+^). Anal. Calc. for C_17_H_15_NO_4_: C, 68.68; H, 5.09; N, 4.71. Found: C, 68.66; H, 5.07; N, 4.75.

#### 1-(3,4-Dimethoxyphenethyl)-5-Hydroxy-2-Methyl-1H-Indole-3-Carboxylic Acid (6e)

Cream powder; yield: (75%); mp: 140 - 142°C. IR (KBr) (ν_max_, cm^-1^): 3280, 2985, 1647, 1244, 831. ^1^H-NMR δ_H_ = 7.41 (d, *J* = 4 Hz, 1H, Ar), 7.33 (d, *J* = 8.0 Hz, 1H, Ar), 6.83 (d, *J* = 8.0 Hz, 1H, Ar), 6.66 - 6.63 (m, 2H, Ar), 6.54 (d, 1H, Ar), 4.30 (t, 2H, CH_2_), 3.69 (s, 3H, OMe), 3.60 (s, 3H, OMe), 2.89 (t, *J* = 8.0 Hz, 2H, CH_2_), 2.39 (s, 3H, Me). ^13^C-NMR *δ*_C_ = 167.28 (C=O), 152.90 (C), 148.90 (C), 147.97 (C), 145.23 (C), 131.14 (C=N), 130.09 (C=N), 128.24 (C), 121.33 (C), 113.18 (CH), 112.25 (CH), 111.58 (CH), 110.93 (CH), 106.07 (CH), 103.15 (CH), 55.97 (CH_3_O), 55.70 (CH_3_O), 44.90 (CH_2_N), 35.06 (CH_2_), 11.75 (CH_3_). MS: m/z (%) = 356 (M+H^+^). Anal. Calc. for C_20_H_21_NO_5_: C, 67.59; H, 5.96; N, 3.94. Found: C, 67.60; H, 5.97; N, 3.92.

#### 5-Hydroxy-2-Methyl-1-(4-Methylbenzyl)-1H-Indole-3-Carboxylic Acid (6f)

White powder; yield: (90%); mp: 176 - 178°C. IR (KBr) (ν_max_, cm^-1^): 3298, 2981, 1729, 1658, 1174, 868. ^1^H-NMR *δ*_H_ = 11.90 (s, 1H, COOH), 8.90 (s, 1H, OH), 7.42 (d, *J* = 2.4 Hz, 1H, Ar), 7.24 (d, *J* = 8.8, 1H, Ar), 7.11 (d, *J* = 8 Hz, 2H, Ar), 6.91 - 6.89 (d, *J* = 8 Hz, 2H, Ar), 6.61 (m, 1H, Ar), 5.36 (s, 2H, CH_2_N), 2.63 (s, 3H, Me), 2.24 (s, 3H, Me). ^13^C-NMR *δ*_C_ = 167.19 (C=O), 153.06 (C), 145.16 (C), 136.88 (C), 134.84 (C=N), 130.78 (C=N), 129.70 (2 CH), 128.11 (C), 126.64 (2 CH), 111.81 (C), 111.11 (CH), 106.00 (CH), 103.60 (CH), 46.07 (CH_2_N), 21.08 (CH_3_), 12.23 (CH_3_). MS: m/z (%) = 318 (M+23). Anal. Calc. for C_18_H_17_NO_3_: C, 73.20; H, 5.80; N, 4.74, Found: C,73.17; H, 5.80; N, 4.77.

#### 1-Cyclohexyl-5-Hydroxy-2-Methyl-1H-Indole-3-Carboxylic Acid (6g)

White powder; yield: (93%); mp: ND. IR (KBr) (ν_max_, cm^-1^): 3268, 2996, 1647, 1244, 864. ^1^H-NMR *δ*_H_ = 11.82 (s, 1H, COOH), 8.85 (s, 1H, OH), 7.48 (d, *J* = 8.8 Hz, 1H, Ar), 7.43 (d, *J* = 2.4 Hz, 1H, Ar), 6.61 (dd, *J* = 8.8, 2.4 Hz, 1H, Ar), 4.34 (m, 1H, NCH, Cy), 2.72 (s, 3H, Me), 1.87 - 1.68 (m, 6H, Cy), 1.49 - 1.37 (m, 4H, Cy, Me). ^13^C-NMR *δ*_C_ = 165.73 (C=O), 152.57 (C), 145.03 (C=N), 129.11 (C=N), 128.59 (C), 113.34 (C), 111.47 (CH), 106.14 (CH), 102.57 (CH), 59.11 (CHN), 30.82 (2 CH_2_), 26.13 (2 CH_2_), 25.19 (CH_2_), 12.57 (CH_3_). MS: m/z (%) = 273 (M+H^+^). Anal. Calc. for C_16_H_19_NO_3_: C, 70.31; H, 7.01; N, 5.12. Found: C, 70.31; H, 7.03; N, 5.10.

#### 1-Benzyl-5-Hydroxy-2-Methyl-1H-Benzo[g]Indole-3-Carboxylic Acid (6h)

White powder; yield: (74%); mp: 179 - 181°C, IR (KBr) (ν_max_, cm^-1^): 3261, 2978, 1662, 1233, 864. ^1^H-NMR *δ*_H_ = 8.25-8.22 (m, 1H, Ar), 8.07-8.03 (dd, *J* = 12.0, 8 Hz, 2H, Ar), 7.33 - 721 (m, 6H, Ar), 7.03-7.01 (m, 1H, Ar), 5.83 (s, 2H, CH_2_N), 2.78 (s, 3H, Me). ^13^C-NMR *δ*_C_ = 173.96 (C=O), 169.10 (C), 148.45 (C=N), 141.60 (C=N), 138.22 (C), 129.33 (CH), 127.57 (C), 126.01(CH), 125.99 (CH), 125.82 (2CH), 123.81 (CH), 123.49 (CH), 123.40 (CH), 122.45 (CH), 122.22 (C), 120.60 (C), 109.12 (C), 103.22 (C), 48.95 (CH_2_N), 11.83 (CH_3_). MS: m/z (%) = 332 (M+H^+^). Anal. Calc. for C_21_H_17_NO_3_: C, 76.12; H, 5.17; N, 4.23. Found: C, 76.14; H, 5.15; N, 4.14.

#### 1-Butyl-5-Hydroxy-2-Methyl-1H-Benzo[g]Indole-3-Carboxylic Acid (6i)

Blue powder; yield: (89%); mp: 200 - 202°C. IR (KBr) (ν_max_, cm^-1^): 3298, 1647, 1248, 864. ^1^H-NMR *δ*_H_ = 12.07 (s, 1H, COOH), 9.71 (s, 1H, OH), 8.28-8.25 (dd, *J* = 8.0, 4.0 Hz, 2H, Ar), 7.76 (s,1H, Ar), 7.61 - 7.51 (dd, *J* =15.6, 7.2 Hz, 1H, Ar), 7.44 - 7.42 (dd, *J* = 15.2, 8.0 Hz, 1H, Ar), 4.52 (t, *J* =7.6 2H, CH_2_N), 2.77 (s, 3H, Me), 1.82-1.77 (m, 2H, CH_2_), 1.48 - 1.38 (m, 2H, CH_2_), 0.96 (t, *J* = 7.2 Hz, 3H, CH_3_). ^13^C-NMR *δ*_H_ = 167.36 (C=O), 148.48 (C), 143.09 (C=N), 126.71 (C=N), 125.28(C), 123.87 (C), 123.38 (C), 123.23 (C), 122.84 (CH), 122.62 (CH), 120.63 (CH), 104.65 (CH), 102.24 (CH), 45.44 (CH_2_N), 31.92 (CH_2_), 19.74 (CH_2_), 14.15 (CH_3_), 12.12 (CH_3_). MS: m/z (%) = 298 (M+H^+^). Anal. Calc. for C_18_H_19_NO_3_: C, 72.71; H, 6.44; N, 4.71. Found: C,72.70; H, 6.43; N, 4.73.

#### 5-Hydroxy-1-(4-Methoxyphenyl)-2-Methyl-1H-Benzo[g]Indole-3- Carboxylic Acid (6j)

Pale purple powder; yield: (91%); mp: 210 - 212°C. IR (KBr) (ν_max_, cm^-1^): 3268, 2963, 1647, 1185, 872. ^1^H-NMR *δ*_H_ = 9.03 (d, *J* = 4 Hz, 1H, Ar), 8.6 (s, 1H, Ar), 8.25 (d, *J* = 8 Hz, 2H, Ar), 8.12-8.04 (m, 3H, Ar), 8.0-7.95 (m, 1H, Ar), 7.77-7.75 (m, 1H, Ar), 4.74 (s, 3H, OMe), 3.24 (s, 3H, Me). ^13^C-NMR *δ*_C_ = 167.60 (C=O), 160.12 (C), 148.70 (C), 143.52 (C=N), 132.09 (C=N), 130.36 (2 CH), 125.85 (CH), 125.02 (C), 124.71 (C), 123.69 (C), 123.37 (C), 122.86 (C), 122.52 (CH), 120.00 (CH), 120.03 (CH), 115.82 (2 CH), 102.15 (CH), 56.02 (CH_3_O), 13.03 (CH_3_). MS: m/z (%) = 348 (M+H^+^). Anal. Calc. for C_21_H_17_NO_4_: C, 72.61; H, 4.93; N, 4.03. Found: C, 72.59; H, 4.92; N, 4.06.

#### 5-Hydroxy-1-(4-Methoxyphenethyl)-2-Methyl-1H-Benzo[g]Indole- 3-Carboxylic Acid (6k)

Gold powder; yield: (79%); mp: 141 - 143°C. IR (KBr) (ν_max_, cm^-1^): 3239, 1666, 1237, 861. ^1^H-NMR *δ*_H_ = 8.43 (d, *J* = 8.4 Hz, 1H, Ar), 8.31 (d, *J* = 8.4 Hz, 1H, Ar), 7.83 (s, 1H, Ar), 7.65 (m, 1H, Ar), 7.46 (m, 1H, Ar), 7.09 (d, *J* = 8.8 Hz, 2H, Ar), 6.89 (d, *J* = 8.8 Hz, 2H, Ar), 4.73 (t, *J* = 7.2 Hz, 2H, CH_2_N), 3.72 (s, 3H, OMe), 3.09 (t, *J* = 7.2 Hz, 2H, CH_2_N ), 2.53 (s, 3H, Me). ^13^C-NMR *δ*_C_ = 167.59 (C=O), 158.55 (C), 148.45 (C), 143.12 (C=N), 130.32 (C=N), 130.22 (2 CH), 126.69 (C), 125.56 (C), 123.97 (C), 123.41 (C), 122.96 (CH), 122.77 (CH), 122.64 (CH), 120.65 (C), 114.46 (2 CH), 102.54 (CH), 55.51 (CH_3_O), 47.33 (CH_2_N), 34.91 (CH_2_), 14.97 (CH_3_), 11.81 (CH_3_). MS: m/z (%) = 376 (M+H^+^). Anal. Calc. for C_23_H_21_NO_4_: C, 73.58; H, 5.64; N, 3.73. Found: C, 73.60; H, 5.62; N, 3.76.

### 2.3. Cytotoxicity Evaluation

#### 2.3.1. Cell Culture

The cytotoxicity of 5-hydroxyindole-carboxylic acids and esters’ derivatives were determined against breast adenocarcinoma, MCF-7, and the results were compared to fibroblast normal cell line, human dermal fibroblasts (HDF). The cell lines were obtained from the Pasture Institute of Iran. The cells were placed on the cell culture flasks with RPMI, filled out with 10% fetal bovine serum and 1% antibiotic solution (10 mg of streptomycin and 10,000 units of penicillin in 0.9% NaCl) in a wet atmosphere of 5% CO_2_ at room temperature in 37°C.

#### 2.3.2. Cell Proliferation Assay

MTT (3-[4,5-dimethylthiazol-2-yl]-2,5-diphenyl tetrazolium bromide) approach is a standard assay to evaluate the inhibitory effect of the compounds (22). The base of the colorimetric assay is the decrease of the yellow color MTT to purple crystals by metabolically smart cells. The live cells have NAD(P)H-dependent oxidoreductase enzymes, decreasing the MTT to purple formazan crystal.

**Figure 4.**
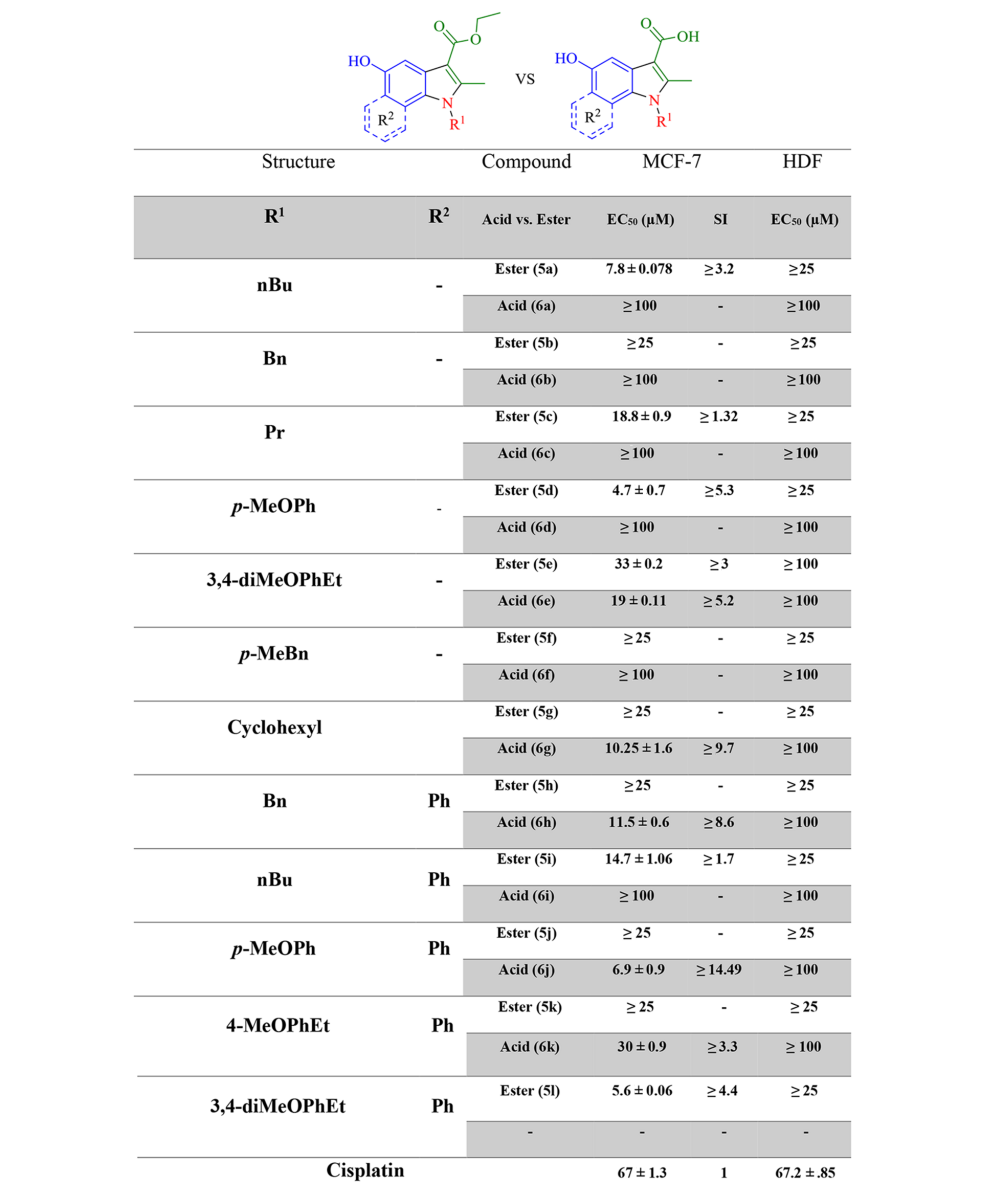
Half-maximal effective concentration in MCF-7 and human dermal fibroblasts for target indole esters (5) and acids (6). HDF, human dermal fibroblasts; EC_50_, half-maximal effective concentration; SI, selectivity index.

For the MTT assay, the cells (10000 cells/well) were cultured in 96-well plates in media and incubated for 24 hours. Then, the solution of each synthesized compound was poured into the wells in three repetitions and six concentrations. The plates were incubated for 24 hours, the culture medium was drained from the wells, and 100 µL of MTT solution in phosphate-buffered saline (0.5 mg/mL) was added to each well and incubated for 4 hours. Then, 100 µL of formazan solvent containing 10% sodium dodecyl sulfate instead of DMSO (23) was added to each well and incubated for 2 hours. Finally, relative cell viability was calculated based on a colorimetric method using a Cell Imaging Multi-Mode Microplate Reader (Biotek, Cytation 3, USA) and recording the absorbance at 570 nm (Figure 4) (24).

### 2.4. Molecular Modeling Study

**Figure 5.**
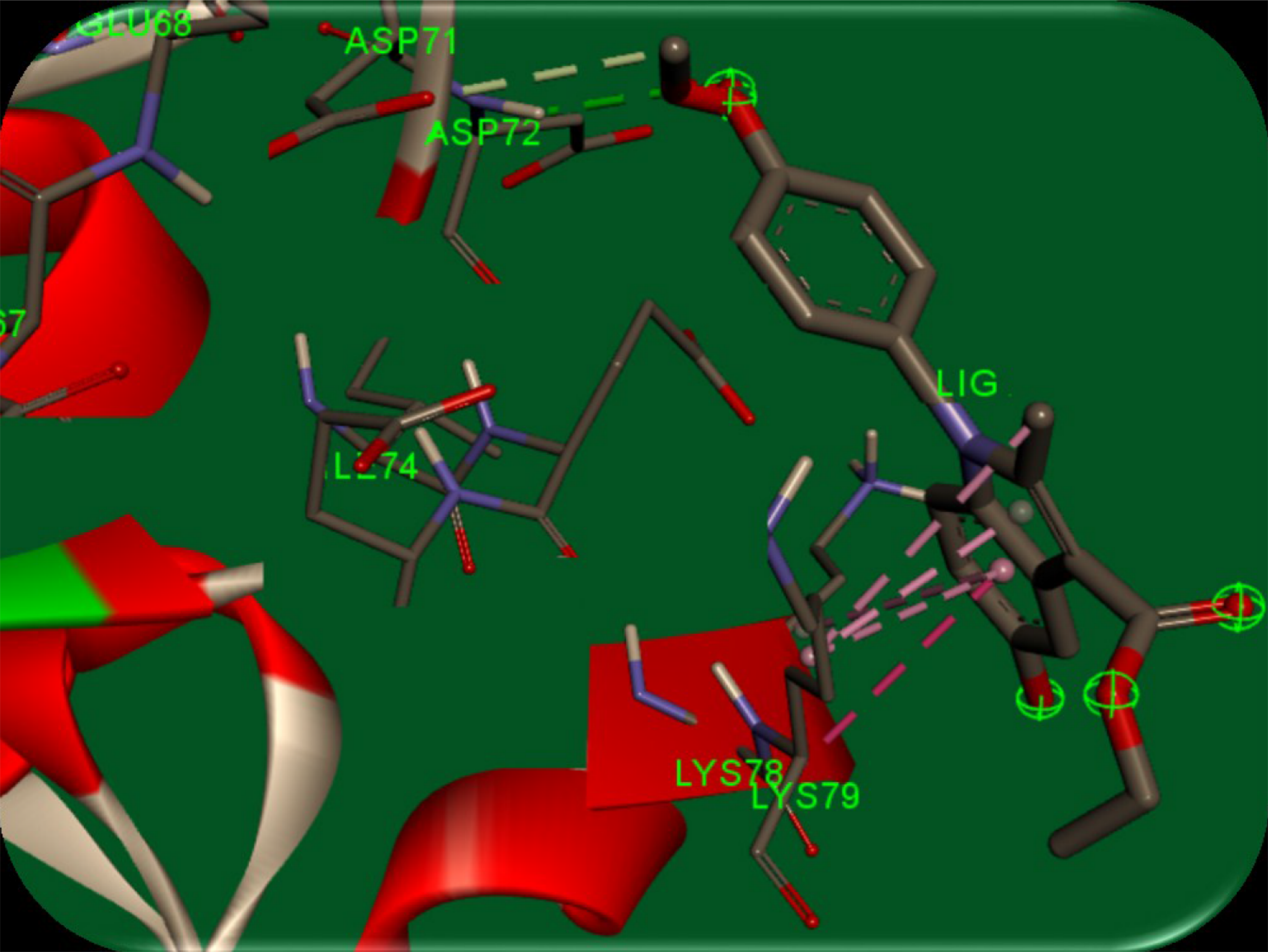
Binding modes of ethyl 5-hydroxy-1-(4-methoxyphenyl)-2-methyl-1h-indole-3-carboxylate (5d) in the active site of survivin protein (PDB entry: 3UIH)

AutoDock software (version 4) was used to perform docking simulations to describe the interactions between synthesized compounds and survivin protein. A stable conformer of the synthesized compounds was docked into the binding pocket of the biological target, survivin (PDB code: 3UIH). The crystal structure was optimized by separating all water molecules from the protein. Kollman charges and polar hydrogens were also added to the protein structure. The output file was converted to PDBQT format using AutoDock 4. In the next step, the energy of the synthesized ligands was minimized through the MM^+^ method using HyperChem 8.0 software. The ligands were converted to PDBQT using AutoDock 4. Then, a grid box of 20-20-20 Å around the protein’s active site was constructed (for more efficacy, the residues with atoms bigger than 7.5 Å were removed from the grid box), and the Lamarckian genetic search algorithm with total runs of 50 was applied. Finally, the most energetically stable conformation was selected. To explain the good potency of 5d that was observed, a molecular modeling study was used. Figure 5 illustrates the complex of human survival (PDB entry: 3UIH) and the results.

## 3. Results and Discussion

### 3.1. Chemistry

#### 3.1.1. Experimental Procedure to Achieve Compounds 5

The general procedure of accessing 5-hydroxyindole 5d: Enamine 3d that achieved with the reaction between the ethyl acetoacetate 1 (3.0 mmol) and 4-methoxy phenylamine 2 (3.0 mmol) under catalytic acid, added dropwise during 15 minutes to a solution of benzoquinone (3 mmol) in CH_2_Cl_2_ (10 mL, reflux), with a CaI_2_ (5 mol%) as a catalyst. The reaction was stirred for an additional hour, and then the mixture was cooled for 3-4 hours. The solid mixture was filtered off and then washed with CH_2_Cl_2_ (10 mL) (25).

#### 3.1.2. General Procedure for Preparation of Compounds 6

To obtain 5-hydroxyindole carboxylic acid 6d, a mixture of indole 5d (0.01 mmol) was melted at 150°C with potassium hydroxide (0.1 mmol) for about an hour and then cooled. Afterward, the least amount of water was added to dissolve the mixture. Glacial acetic acid was added dropwise to the solution to precipitate the almost pure product. Analytical samples were prepared by crystallization from dilute ethanol.

### 3.2. Biological Evaluations

**Figure 6.**
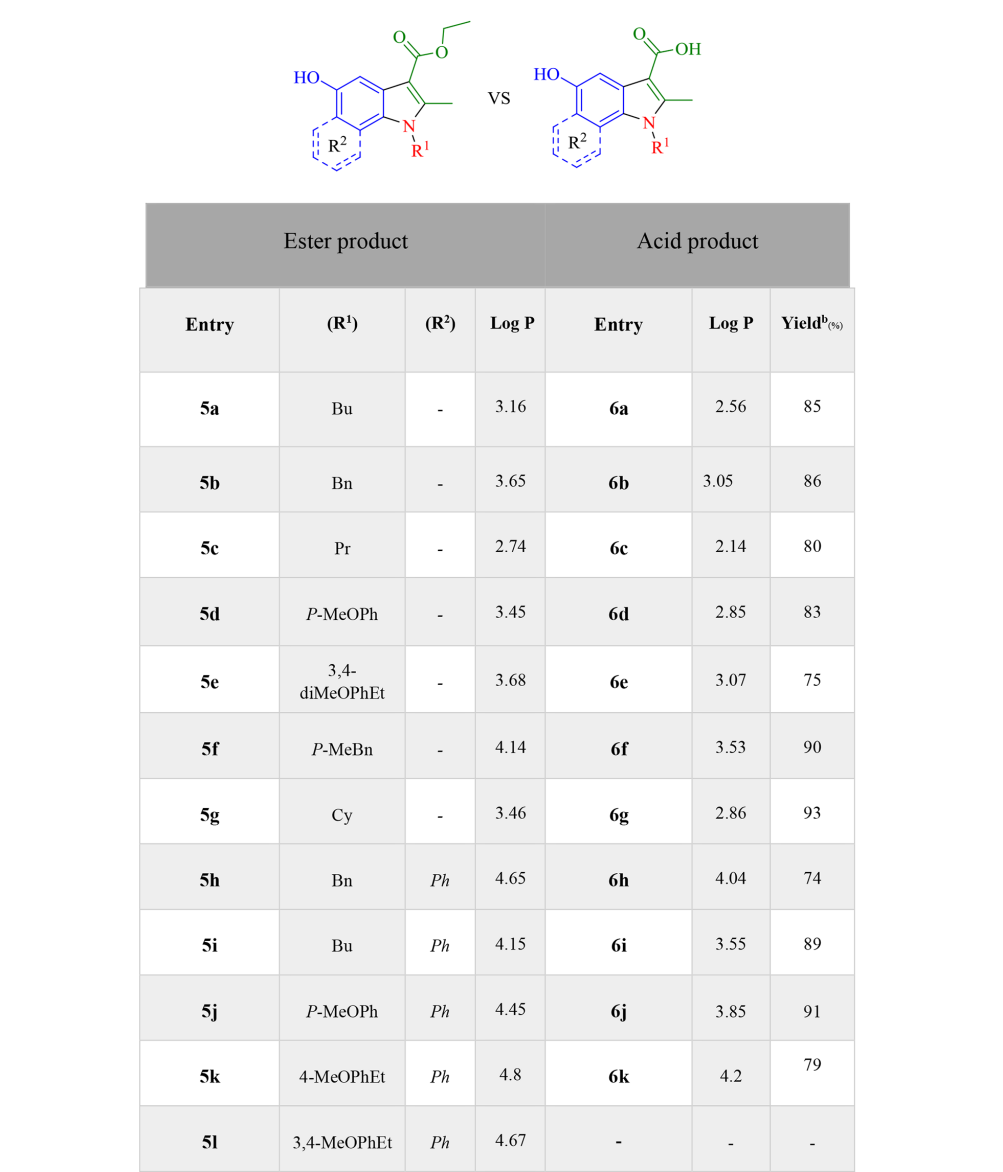
Comparison of log P for ester and acid derivatives of 5-hydroxyindole derivatives

With the MTT assay, the cytotoxic effects of compounds, which were selected via docking studies, were evaluated against the MCF-7 breast cancer cell line. Furthermore, the cytotoxicity was assessed on the normal HDF line. The antiproliferative activities of selected compounds were compared to cisplatin at six concentrations within 31.25 - 1000 μM on MCF-7 and normal fibroblast cells. As depicted in Figure 4, all the selected compounds showed cytotoxic effects on the MCF-7 cell line, compared to cisplatin. 5a, 5d, 5l, and 6j derivatives from esters and acids showed the highest potency against the MCF-7 cell line among the other compounds. According to the structural similarity, combinations, including the *p*-methoxy phenyl group, substituted in R^2^ generally had better potency than the benzyl or *p*-methyl benzyl group. Moreover, the results revealed that electron-donating groups in the *para* part of the indole ring had better cytotoxic activity than compounds without them. Because, on the one hand, the solubility of new synthetic compounds was very important in the cancer biological test mediums, and on the other hand, the amount of hydrophilicity or hydrophobicity of compounds must be evaluated, log P was measured with the ChemDraw software to compare acid and ester products and then indicated that not only the solubility improved but also the amount of log P was within the allowed range of Lipinski’s rule of five (log P < 5) (Figure 6).

### 3.3. Docking Study

It is speculated that the *para* methoxy group in the 5d ligand has hydrogen bondings between Asp71 and Asp72 residues. Furthermore, the 5-hydroxy indole group and the carboxylate group form π-π stacking interaction between Lys79 residue in the active site. Green bonds indicate hydrogen bonds between Asp71 (with 3.50 Å distance) and Asp72 (with 2.11 Å distance) amino acids with *p*-methoxy phenyl in 5d ligand, and pink bond is a π-π stacking interaction between the 5d indole and residue Lys79 with 3.58 Å distance. The results indicated the minimal cytotoxic effect of the compounds at a concentration of 100 μM against normal HDF cells. A molecular modeling study on compound 5d showed proper orientation with the survivin protein active site (Figure 5).

## 4. Conclusions

In summary, various *N*-substituted 5-hydroxyindole-3-carboxylic acids and esters were designed and synthesized based on targeted structural modification of the UC-112 and PCA lead compounds. The structure-activity relationships were investigated by changing the amine groups inserted in the indole ring, changing the ester bond to the acid bond. Ultimately, their log P was checked to ensure that the hydrophobic properties did not decrease much. These small molecules indicated a significant cytotoxic effect on the MCF-7 cell line and low toxicity on normal fibroblast cells. The aforementioned results showed a good correlation with the docking study. The most potent investigated compound, 5d, was shown to exhibit well drug-like properties. These novel small-molecule inhibitors of survivin are currently being developed to produce more selective and effective survivin inhibitors. However, further studies are needed to find the exact mechanism and possible off-targets.

## Data Availability

The data presented in this study are uploaded during submission as a supplementary file and are openly available for readers upon request.
